# Analysis of Transcriptome and miRNAome in the Muscle of Bamei Pigs at Different Developmental Stages

**DOI:** 10.3390/ani10071198

**Published:** 2020-07-15

**Authors:** Guofang Wu, Lin Ma, Lei Wang, Jiping Zhou, Yuhong Ma, Chen Yang

**Affiliations:** Stake Key Laboratory of Plateau Ecology and Agriculture, Qinghai Academy of Animal Science and Veterinary Medicine, Qinghai University, Xining 810016, China; letitbe521@163.com (G.W.); malin920412@163.com (L.M.); mkyzhjp@126.com (J.Z.); 2004990028@qhu.edu.cn (Y.M.); 1982990042@qhu.edu.cn (C.Y.)

**Keywords:** Bamei pig, transcriptome, differentially expressed (DE) genes, miRNA, *longissimus dorsi* muscle

## Abstract

**Simple Summary:**

The pigs is the most popular agricultural animal in the world. Muscle growth—which has the highest economic value in pigs—can be regulated by multiple genes and involves complex regulatory mechanisms. It is necessary to understand the dynamics of muscle transcriptome during development to understand the muscle development mechanism. However, the genes and miRNAs that play regulatory roles underlying differences in the meat quality of pigs remain unclear. In the current study, qRT-PCR, miRNA-Seq, and RNA-Seq were applied to analyze and verify muscle tissues of pigs from three different developmental stages and screened genes, miRNAs and pathways related to pig muscle development. This study focused on analyzing the mechanisms of muscle development and uncover the development differences in muscle from embryo to adult.

**Abstract:**

The growth of skeletal muscle involves complex developmental processes that play an important part in the determinization of pork quality. The investigation of skeletal muscle mRNA or miRNA profiles is especially important for finding molecular approaches to improve meat quality in pig breeding. Therefore, we studied the transcriptome (mRNA and miRNA) profiles of skeletal muscle with RNA-Seq in three developmental stages of pigs: 65-day embryonic (E65), postnatal 0 days (natal) and 10 months (adult). We found 10,035, 9050 and 4841 differentially expressed (DE) genes for natal vs. E65, adult vs. E65 and adult vs. natal, 55, 101 and 85 DE miRNA for natal vs. E65, adult vs. E65 and adult vs. natal, respectively. In addition, the target genes of DE miRNA that was in a negative correlation with the corresponding miRNA in the same comparison group were selected for enrichment analysis. Gene Ontology terms were mainly classified into developmental processes. Pathway analysis revealed enrichment in the Rap1 signaling pathway, citrate cycle and oxidative phosphorylation and carbon. Finally, RT-PCR was employed for validating the level of expression of 11 DE miRNA and 14 DEGs. The transcriptome profiles of skeletal muscle from the different developmental stages of the Bamei pigs were obtained. From these data, hundreds of DE miRNA and mRNA, and the miRNA–mRNA regulatory network can provide valuable insights into further understanding of key molecular mechanisms and improving the meat quality in pig breeding.

## 1. Introduction

Skeletal muscle is the primary meat production tissue of pigs and plays a central role in pig activity. However, the growth of skeletal muscle is a complicated and precisely regulated process, and the potential regulatory mechanisms in different meat quality are still unclear [1,2]. Therefore, the insight of molecular mechanisms could provide a solid foundation for understanding the characteristics of different developmental stages in porcine skeletal muscle.

Pigs are one of the most critical livestock species because of their economic importance in the livestock industry. The Bamei breed is a native and special pig breed mainly raised in northwestern China. Bamei pigs have genetic stability and good meat quality characteristics due to the process of artificial and natural selection which occurred over long period of time [3].

In recent years, with the evolution of sequencing technology, increasing sequencing is used in the identification of differential expression. A few transcriptome studies had been done to review gene expression or genetic variation profiling in livestock [4,5,6,7]. There are many studies on mRNA and long non-coding RNAs (lncRNAs) levels in the muscle from different pig breeds [8,9,10,11]. In 2018, the transcriptome data of *longissimus dorsi* muscle (LDM) between two different intramuscular fat content pigs were compared using transcriptome sequencing. The results showed that the *myostatin*, SOCS box containing 2 (ASB2) and ankyrin repeat, and ankyrin repeat domains 1 and 2 (ANKRD1/2) could affect the development of porcine skeletal muscle [9]. Xu et al. uncovered a set of DE genes (DEGs), which are potentially associated with myofiber development in the skeletal muscle fiber phenotypes between Northeast Min pigs and the Changbaishan wild boar [8]. Seventy-one significant DEGs (15 for LDM and 56 for semimembranosus muscles) were found for the Pulawska and Polish Landrace breeds [12]. RNA sequencing (RNA-Seq) and miRNA sequencing were also used to find out the relation of transcriptome profiles of skeletal muscle in two pigs breeds at five developmental stages of gestation [13,14]. A total of 1317 and 691 DEGs were detected in TC and YK pigs and 23, 30, 12, 6, and 30 breed- DE miRNA was identified between YK and TC at five different developmental stages. E1A binding protein P300 (*EP300*), cyclin-dependent kinase 14 (*CDK14*), insulin receptor substrate 1 (*IRS1*), tensin homolog (*PTEN*) and phosphatase, *Myosin 9A,* protein phosphatase 1 catalytic subunit gamma (*PPP1CC*) and miR-499-5p were identified as the important molecular markers for deciphering the developmental differences in two pigs breeds. In 2019, 12 DE miRNA in muscle were found between Yorkshire and seven indigenous breeds [15]. Shang et al. identified 20 genes that were related to muscle fiber formation in pigs using transcriptome and proteome data [16]. In addition, four genes (including *MYOD1*, *PPARD*, miR-423-5p, and miR-874) were identified to be associated with muscular proliferation by mRNAs-lncRNAs-circle RNAs regulatory networks analysis [17]. However, there are few studies that have been conducted on the skeletal muscle of Chinese native pigs as research objects. 

In addition, muscle development is associated with the developmental stage. The prenatal and postnatal muscle growth are significant sources of gene mining for the molecular events that control adult muscle traits. The integrated analysis of miRNA and mRNA transcriptome data about skeletal muscle is not commonly reported.

To well recognize the transcriptional regulatory mechanism of skeletal muscle, the transcriptome of LDM was compared in Bamei pigs across embryonic (65 days, E65), postnatal (0 days, natal) and adult (10 months of age) stages of life, which are representative of the major developmental stages in Bamei pigs. Next-generation sequencing was used to integrate mRNA and miRNA data analysis. This procedure allowed us to identify important regulatory elements and find several candidates genes that could be used for selection in pigs. These findings could support to explain the difference in the transcriptional pattern of muscle development and growth.

## 2. Materials and Methods

### 2.1. Animal and Longissimus Dorsi Muscle Tissue Collection

All the protocols followed in these experiments were approved by the Ethical Committee of the Qinghai Academy of Animal Science and Veterinary Medicine. Nine healthy female Bamei pigs were grouped into three developmental stages (three replicates for each stage): E65, natal and adult. The E65 and natal pigs were taken from different sows. The E65 were taken from one pregnant sow at 65 days after insemination. For the natal group, all piglets were inbred and were from the same sow and same boar. The pregnant sow was raised in healthy condition and the weight of sow is 120 kg. All pigs were quarantined. All the sows are fertilized by natural mating. The natal pigs were slaughtered just after they were born, so they did not experience weaning and did not have diarrhea symptoms. However, for the adult group, antibiotics were used during weaning. adults were fed ad libitum up to 90 kg of body weight. adults were slaughtered at an average age of ten months. All animals were slaughtered following the procedures of the institutional animal care and use committee. LDM samples were collected from the Huzhu breeding pigs farm (Qinghai province in China).

### 2.2. RNA Extraction and Quality Assessment

RNAiso Plus was employed for the isolation of total RNA from LDM tissues following the prescribed procedures and specifications. The Qubit^®^ Assay Kit was used with the Qubit^®^ 2.0 Fluorometer (Life Technologies, Carlsbad, CA, USA) for selective and accurate quantification of the RNA. The RNA purity was determined using a NanoPhotometer^®^ (IMPLEN, Westlake Village, CA, USA). The Bioanalyzer 2100 system (Agilent Technologies, Santa Clara, CA, USA) was employed for measuring the RNA integrity number (RIN). The RIN values, 28S/18S values and A_260/280_ ratios of all pig specimens are depicted in Table S1 of the Supplementary Materials.

### 2.3. Construction of cDNA Library and Illumina Sequencing

The mRNA was isolated from total RNA and fragmentation was generated. M-MuLV Reverse Transcriptase (RNase H) and random primer were employed to replicate the first-strand cDNA while the second-strand cDNA was replicated by RNase H and DNA Polymerase I. The NEBNext adaptor having a structure like hairpin was joined to equip for hybridization after the DNA fragments adenylation at 3′ ends. After that, the qualities of the library were assessed using a Bioanalyzer 2100 system. The Illumina HiSeq platform was employed for the sequencing of the nine RNA-Seq library preparations and as a result, 150 bp paired-end reads were gained.

### 2.4. Construction of Small RNA Library and Illumina Sequencing

First of all, the total RNA was pooled in the natal, E65 and adult for replicates. The 3′ and 5′ adaptors were joined to 3′ and 5′ ends of small RNA (sRNA) and the first-strand cDNA was replicated. The products obtained from PCR were purified via polyacrylamide gel and DNA fragments of 140–160 bp were collected. The quality of the library was evaluated on the Bioanalyzer 2100 equipped with High Sensitivity Chips of DNA. The Illumina HiSeq platform was employed for sequencing of the library preparations and 50 bp single-end reads were gained. All sRNA datasets were submitted to the Sequence Read Archive of the NCBI database (accession numbers: SRR10390626, SRR10390625 and SRR10390624).

### 2.5. Analyses of RNA-Seq Data

Python scripts were used to remove reads comprising an adapter, low-quality reads and holding more than 10% N from raw reads. The cleaned and high-quality data were further subjected to downstream analysis.

All clean reads were associated with the pig’s reference genome *Sus scrofa v10.2* with TopH at2, and only two mismatches are allowed [18]. After that, novel transcripts were gathered employing Cufflinks (v2.2.1) with the default values of all the parameters [19]. The HTSeq (v0.6.1) was used to calculate the reads numbers which mapped to each gene [20] and subsequently fragments per kilobase of the exon model per million mapped reads (FPKM) value of each gene were calculated [21].

The R package DESeq (v1.18.0) was used to obtain DEGs of three groups (Each group consisted of three biologic replicates) [22]. DESeq affords statistical procedures based on the negative binomial distribution to measure the differential expression in digital gene expression data. The approach of Benjamini and Hochberg was followed for adjusting the *p*-values so the false discovery rate (FDR) can be controlled effectively [23]. For padj-values < 0.05 were defined as DEGs.

DEGs were marked up via the Kyoto Encyclopedia of Gene and genome (KEGG) and Gene Ontology (GO) databases for investigating the core function of the genes. The GO database was used to predict the function of the genes, including the cellular component, biologic processes and molecular function [24]. GO enrichment analysis of DEGs were carried out with the GOSeq package [25]. For padj-values < 0.05, it was considered that DEGs significantly enriched the GO terms [26]. The KOBAS (v2.0) was employed for testing the DEGs statistical enrichment in the pathways of KEGG [27].

### 2.6. Analyses of sRNA-Seq Data

Python scripts were used to remove reads comprising adapter contaminants, low-quality reads, containing poly-C or G or T or A, the insert tag or without 3′ adapter and holding more than 10% poly-N from raw reads. Then, 18–35 nt of length were chosen to be subjected to downstream analysis.

The Bowtie was used to align the sRNA reads without a mismatch to the pigs reference sequence [28]. The known miRNA was searched by the mapped sRNA reads. The srna-tools-cli and miRDeep2 were employed for obtaining the potential miRNA using the miRbase 20.0 as a reference [29,30]. The miRNA counts were measured using Python scripts. To remove reads replicating from repeat sequences, protein-coding genes, sRNA and transfer RNA, small nuclear RNA and ribosomal RNA were aligned to the Rfam database, RepeatMasker (Smit et al. http://repeatmasker.org), and those types of data from selected species [31,32].

The novel miRNA was predicted with miRDeep2 and miREvo software using the distinguished hairpin structure of miRNA precursor [29,33]. Meanwhile, the miRNA counts were calculated and the levels of miRNA expression were evaluated by transcript per million (TPM) [34] following the below given formula:

Normalized expression = (mapped read count/total reads) × 1,000,000.

DEGSeq R package was used to obtain the differential expression data of three groups [35]. The *p*-values were adjusted by padj [36]. The padj < 0.01 and |fold change| > two were assigned as significant difference in gene expression.

### 2.7. Integrated Analysis of DE Genes and miRNA

The miRanda software was used for predicting the target genes for revealing the biologic functions of miRNA [37]. As miRNA tends to downregulate their target genes expression, DE target genes that were negatively correlated with that of the corresponding miRNA in the same comparison group were selected for further investigation.

The DE miRNA target gene was passed through the analysis of GO enrichment based on the Wallenius non-central hyper-geometric distribution using GOSeq [25]. The predicted target genes were also annotated using the KEGG database [26]. Mainly signal transduction pathways for the target genes were identified by KEGG pathway analysis. The KOBAS (v2.0) was used to analyze the statistical enrichment of the target gene in the pathways of KEGG [27].

### 2.8. Validation of Sequencing Results by Quantitative Real-Time PCR (qRT-PCR)

For the quantitative determination of the reliability of sequencing data, qRT-PCR was carried out to test expression levels of 14 DEGs and 11 DE miRNA which was randomly selected. There were nine tissues used for the experiments: E65-1, 2, 3, natal-1, 2, 3 and adult-1, 2, 3 (each stage consists of three biologic replicates).

For mRNA, the GAPDH was selected as the internal controls. Reverse transcription-PCR was implemented to generate cDNA by the 5× All-in-One RT MasterMix with AccuRT Genomic DNA Removal Kit (abm) following the manufacturer’s recommendations. qRT-PCR was carried out using the EvaGreen 2× qPCR MaterMix (abm) on a Bio-Rad CFX96 RT-PCR system. Each 10 μL qRT-PCR system contained 5 μL EvaGreen 2× qPCR MasterMix, 0.6 μL cDNA, 3.8 nuclease-free H_2_O, 3-μmol PCR forward primer and 3-μmol reverse primer. The following procedures were used for qRT-PCR: pre-denaturation for 10 min at 95 °C, 40 cycles of 95 °C for 15 s, 60 °C for 1 min.

The U6 was selected as the internal controls for miRNA. Reverse transcription-PCR was carried out for the synthesis of cDNA using the kit of miRNA cDNA Synthesis integrated with Poly(A) polymerase tailing (abm). qRT-PCR was performed using the EvaGreen miRNA qPCR MasterMix (abm) on the Bio-Rad CFX96 RT-PCR. Each 20 μL qRT-PCR system detained 10 μL EvaGreen miRNA MasterMix, 1 μL cDNA, 7.8 sterile H_2_O, 6-μmol PCR forward primer and 6-μmol reverse primer. The following procedures were used for qRT-PCR: pre-denaturation for 10 min at 95 °C, 40 cycles of 95 °C for 10 s, 63 °C for 15 s, 72 °C for 8 s.

Experiments in this research work were carried out in triplicate where necessary and all the primers are given in Tables S2 and S3 of the Supplementary data file. The 2^−△△Ct^ method was used to measure the levels of relative expression and expression differences were analyze by Student’s t-test [38]. For *p*-value < 0.05 was considered statistically significant.

## 3. Results

### 3.1. Summary of RNA Sequencing Data Mapping and Annotation

**Figure 1:**
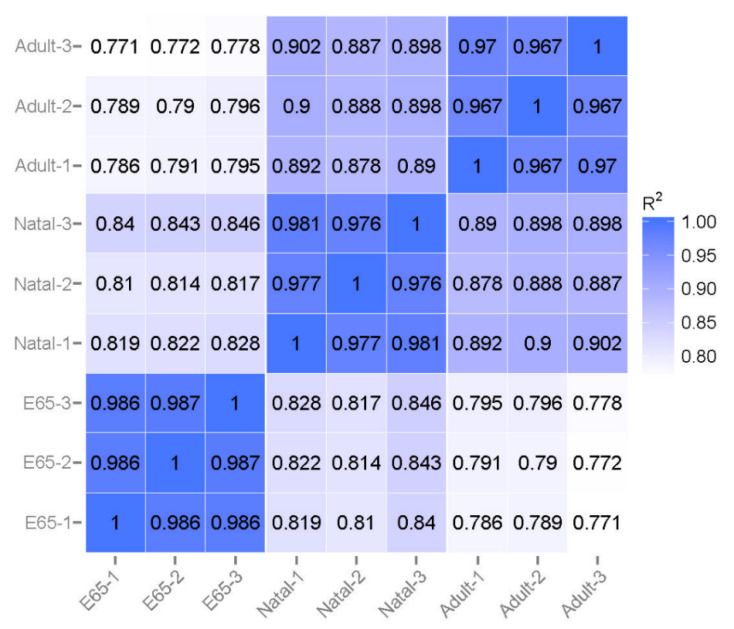
Correlation of gene expression levels among the samples. Pearson correlation coefficients are shown within each cell.

Overall, nine cDNA libraries were constructed from LDM samples of E65, natal and adult groups (*n* = three from each). On average, 97.39% of all sequences passed quality control, of which approximately 71.63–75.25% sequences mapped to the reference genome (Supplementary Table S4). Additionally, 81.4% of reads were mapped to exons, 11.8% to intergenic regions and 6.8% to intronic regions. About 4% of 24,063 annotated genes were expressed > 60 FPKM (Supplementary Table S5); where approximately 12% were expressed between 1–3, 20% between 3–15, and 14% between 15–60 FPKM while the rest of gene expressed less than 1%. The results reliability was ensured by analyzing pairwise correlation based on the normalized FPKM values amid any two replicates in each group. The R values were no less than 0.96 (Figure 1), indicating a high reproducible sample selection.

**Figure 2:**
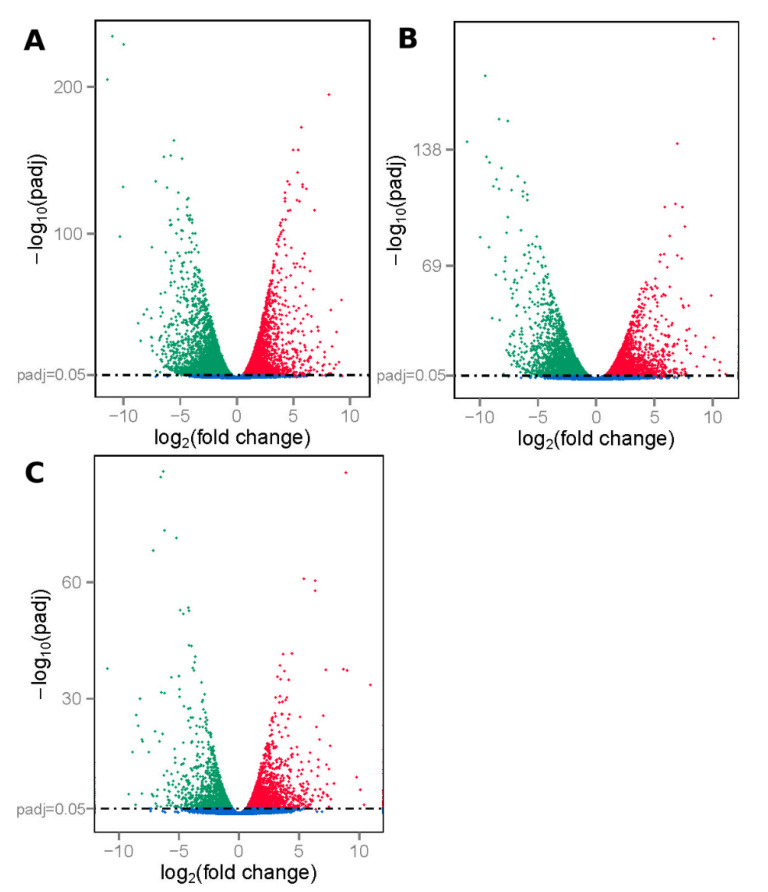
Apex levels of gene expression in (**A**) natal pigs vs. 65-day embryonic (E65) pigs, (**B**) adult pigs vs. E65 pigs and (**C**) adult pigs vs. natal pigs. The padj of 0.05 is represented by a horizontal line. The DEGs downregulated and upregulated were represented by red points and green points, respectively.

**Figure 3:**
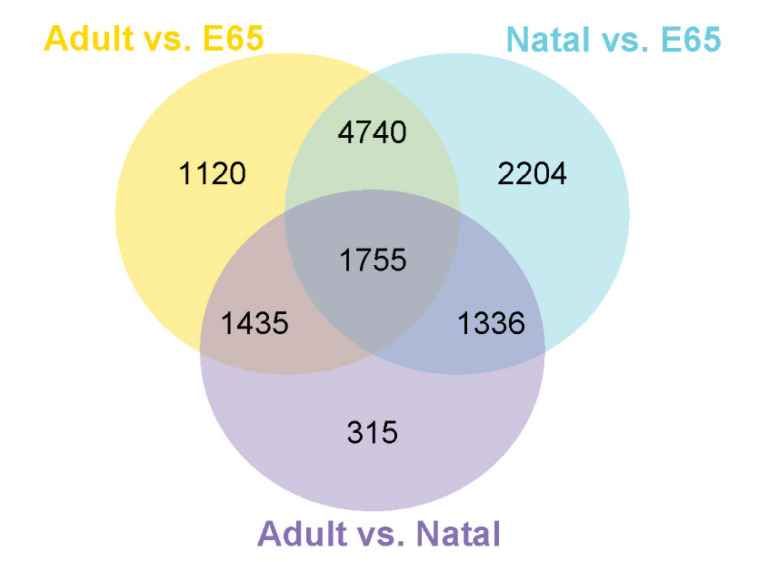
Presentation of the DE genes (DEGs) numbers amid the three groups of pairwise correlation in Venn diagram form.

To better study the differences in gene expression patterns among muscle of E65, natal and adult pigs, we used DESeq to identify DEGs between any two muscle samples (natal vs. E65, adult vs. E65 and adult vs. natal). A total of 24,063 DEGs were detected in three groups of pairwise comparisons. Among these, the number of DEGs in natal vs. E65, adult vs. E65 and adult vs. natal were 10,035 (4.64 upregulated in natal and 5171 in E65), 9050 (4400 upregulated in adult and 4650 in E65) and 4841 (2579 upregulated in adult and 2262 in natal), respectively (Figure 2). The 1755 common DEGs were identified in the three comparisons. Except for 1755 genes, the natal vs. E65 and adult vs. E65 showed 4740 common DEGs, the natal vs. E65 and adult vs. natal showed 1336 common DEGs, while the adult vs. E65 and adult vs. natal showed 1435 common DEGs (Figure 3). Furthermore, genes with fold change > two and *p*-value (padj) < 0.05 were also identified. A total of 6519, 6833, and 3348 genes are expressed differentially in natal vs. E65, adult vs. E65 and adult vs. natal, respectively (Supplementary Table S6).

**Figure 4:**
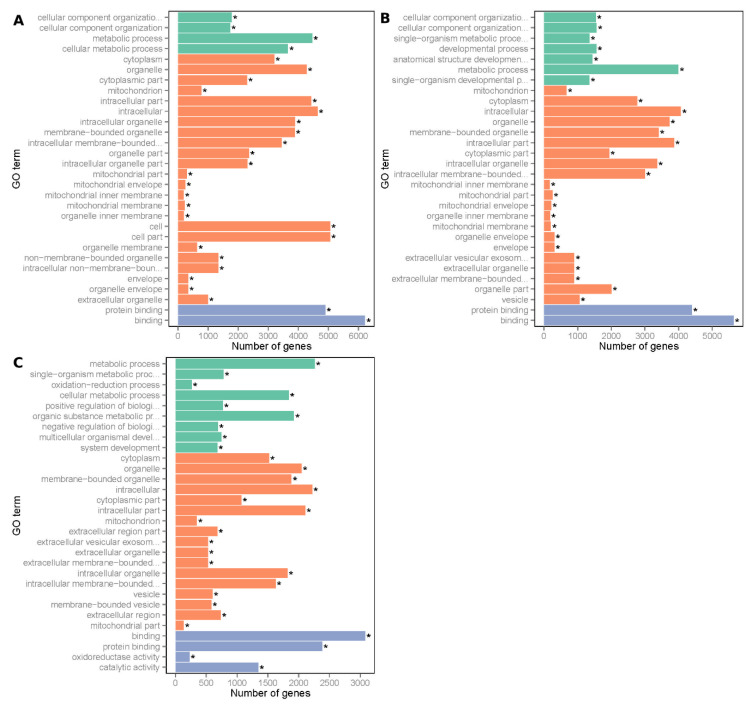
Top 30 of gene ontology (GO)-enrichment for DEGs from three groups of pairwise comparisons. (**A**) Natal pigs vs. E65 pigs; (**B**) adult pigs vs. E65 pigs and (**C**) adult pigs vs. natal pigs. The *x*-axis presents the number of DEGs in a category. The *y*-axis shows the specific GO term. The green histogram represents the biologic process, the orange histogram represents cellular component and the purple histogram represents the molecular function. The asterisk (*) indicates a threshold of padj < 0.05.

The DEGs in the muscle of natal vs. E65, adult vs. E65 and adult vs. natal were annotated. The top 30 GO terms of the three sets are presented in descending order based on their Richness factor (Figure 4). The 843 GO terms, including protein binding, biogenesis or cellular component organization and cytoplasm were found to be significantly enriched with the DEGs of natal vs. E65. Similarly, the 602 GO terms, including mitochondrion, protein binding, and cellular component were found to be significantly enriched with the DEGs of adult vs. E65. The DEGs of adult vs. natal were found significantly enriched in 362 GO terms, including cytoplasm, metabolic process and protein binding.

**Figure 5:**
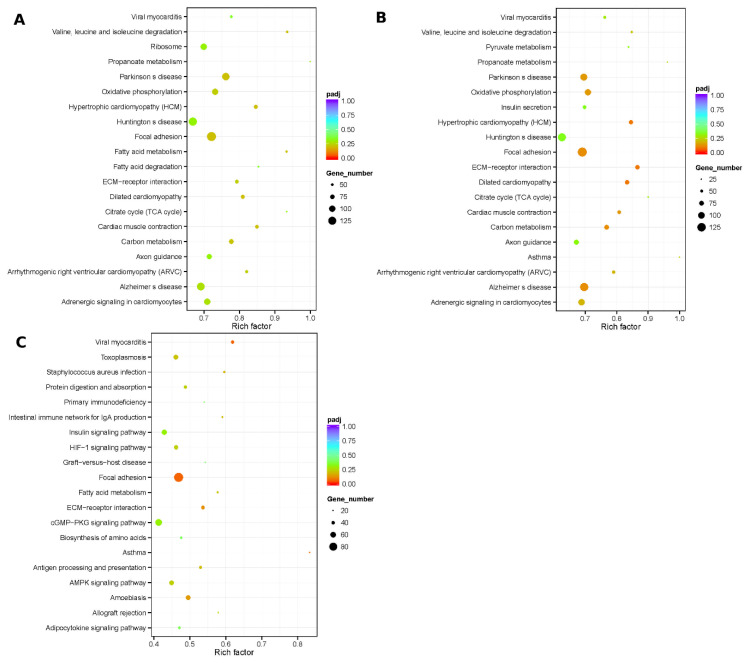
Top 20 of KEGG pathways enrichment for DEGs from three groups of pairwise comparisons. (**A**) Natal pigs vs. E65 pigs; (**B**) adult pigs vs. E65 pigs and (**C**) adult pigs vs. natal pigs. The *x*-axis presents a rich factor (rich factor = amount of DEGs enriched in the pathway/pathway/number of annotated genes). The *y*-axis shows the specific pathway.

KEGG database was used to annotate the pathway of DEGs. The results of pathway enrichment exhibited that the DEGs of natal vs. E65 were enriched in the 28 pathways significantly, including cardiac muscle contraction, ribosome and propanoate metabolism; for DEGs of adult vs. E65 and adult vs. natal, there were no statistically significantly enriched pathways (Figure 5).

### 3.2. Summary of sRNA Sequencing Data Mapping and Annotation

To identify the sRNA involved in muscle proliferation and differentiation of pig, the sRNA libraries were developed from the total RNA of LDM at E65, natal and adult stages. We obtained 10,361,559, 13,504,476 and 11,291,705 clean reads from the E65, natal and adult pig library after filtered (Supplementary Table S7). It was observed from the length distribution results that most of the reads fall in the range of 21 to 23 nt. The reads with 22 nt lengths in the total were 73.85%, 67.50% and 85.92% for the E65, natal and adult stage, respectively (Supplementary Figure S1). This was followed by the use of the Bowtie for aligning all clean reads against the pigs genome [28] and the range of 92.00–96.73 reads showed accurately matched to the genome of the pigs (Supplementary Table S7). The miRNA was measured as one of the largest numbers of RNA species in all libraries, representing 73.92% of the E65, 68.96% of the natal and 77.21% of the adult. The genome-matched sRNA reads were then clustered into several RNA categories in the three libraries (Supplementary Table S8). Additionally, a high proportion of sRNA was classified as other (unknown) RNA (23.63% for E65, 27.46% for natal and 21.95% for adult).

For the identification of conserved miRNA in pig, all reads of sRNA were mapped to known miRNA in the miRBase 20.0 database [30]. There were 28 novels and 301 known miRNAs detected in the muscle tissue of pig. Of 329 identified miRNA, 243 miRNAs were expressed in all samples, whereas 8, 14 and 2 miRNAs were specifically expressed in E65, natal and adult, respectively.

A comparison amid any two stages resulted in DE miRNA. For natal vs. E65, 55 miRNAs were considered as DE miRNA, including 23 downregulated and 32 upregulated genes. Similarly, the adult vs. E65 showed that 101 miRNAs were DE, including 68 downregulated and 33 upregulated ones. In a comparison of the adult and natal, there were 52 downregulated and 33 upregulated found with a total of 85 miRNA DE genes.

### 3.3. Identification of Potential Target Genes of DE miRNA

The potential target genes of DE miRNA were predicted with miRanda software. The probable 9259 genes were identified that may be potential targets of the 134 DE miRNA in the E65, natal and adult pigs [37].

### 3.4. Integrated Analysis of DE Genes and miRNA

To further reveal how miRNAs regulate mRNA, we integrated the DE miRNA with DEGs between the different developmental stages to predict miRNA–mRNA interactions. Nearly 5903 DEGs were observed that may be possible targets of the 134 DE miRNA. When testing the association between the targets and the miRNA and, only the negatively associated mRNA- miRNA pairs have biologically meaningful. Hence, these pairs were measured as potential mRNA- miRNA interactions. A total of 5388 target genes negatively showed interactions with miRNA. For natal vs. E65, there were potentially 1898 down-mRNA and 1805 up-mRNA regulated by 32 up-miRNA and 23 down-miRNA, respectively; for adult vs. E65, there were 1679 down-mRNA and 2054 up-mRNA regulated by 33 up-miRNA and 68 down-miRNA, respectively; and for adult and natal, there were 855 down-mRNA and 1130 up-mRNA regulated by 33 up-miRNA and 52 down-miRNA.

The predicted DE targeted genes of miRNA were further analyzed by the KEGG pathway and GO analysis. The GO study depicted that the genes of the downregulated target in the natal and E65 were remarkably overrepresented in 498 GO terms (padj < 0.01), including protein binding, anatomic structure development and developmental process. The upregulated target genes in natal vs. E65 were significantly overrepresented in 328 GO terms (padj < 0.01), including cytoplasm, cellular metabolic process and catalytic activity. For adult vs. E65, genes of downregulated targets were overrepresented in 360 GO terms (padj < 0.01), including protein binding, organelle and cellular component organization. The genes of upregulated targets were overrepresented in 436 GO terms (padj < 0.01), including mitochondrion, metabolic process and catalytic activity. For adult vs. natal, genes of downregulated targets were overrepresented in 121 GO terms (padj < 0.01), including intracellular, binding and organic substance metabolic processes. The genes of upregulated targets were overrepresented in 279 GO terms at padj < 0.01), including positive regulation of the biologic process, extracellular region part and catalytic activity. The top 60 GO terms of six sets are displayed in Supplementary Figure S2.

Analysis of the pathway revealed that the natal vs. E65 downregulated target genes were present in a large proportion in the Rap1 signaling pathway (padj < 0.01). The upregulated target genes in natal vs. E65 were remarkably present in large numbers in nine pathways (padj < 0.05), including the citrate cycle (TCA cycle), oxidative phosphorylation and carbon metabolism. For adult vs. E65, genes of the downregulated targets were overrepresented in ECM–receptor interaction and cell cycle (padj < 0.01). The genes of the upregulated targets were overrepresented in six pathways (padj < 0.01), including carbon metabolism, oxidative phosphorylation and citrate cycle (TCA cycle). Statistically, there were no significantly enriched pathways in adult vs. natal for the genes of the downregulated targets. However, the genes of the upregulated targets were significantly presented in six pathways (padj < 0.05), including intestinal immune network and antigen processing for IgA production, primary immunodeficiency and presentation. The top 20 pathways of the six groups are shown in Supplementary Figure S3.

### 3.5. Validation of Sequencing Data by qRT-PCR

**Figure 6:**
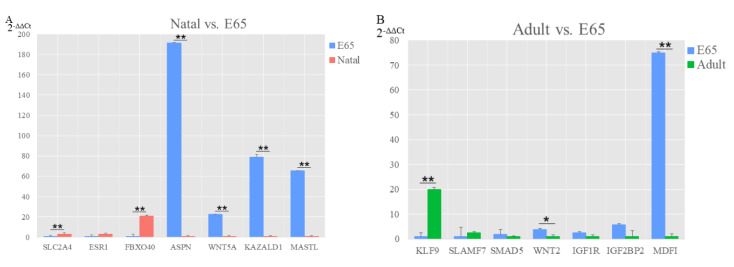
Validation of DEGs from three groups of pairwise comparisons by qRT-PCR. (**A**) Natal pigs vs. E65 pigs and (**B**) adult pigs vs. E65 pigs. Data presented in the *y*-axis indicate genes expression as determined by qRT-PCR. * *p*-value < 0.05; ** *p*-value < 0.01.

**Figure 7:**
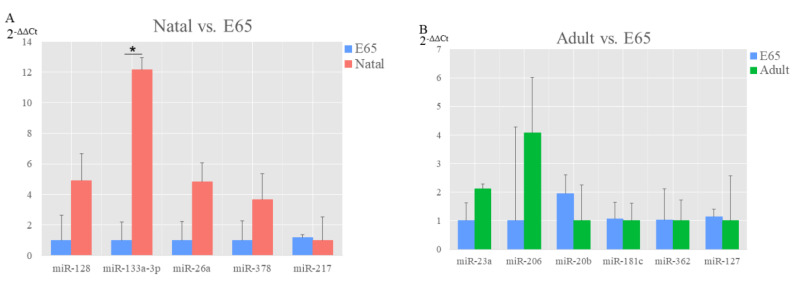
Validation of differentially expressed (DE) miRNA from three groups of pairwise comparisons by qRT-PCR (**A**: natal vs. E65 and **B**: adult vs. E65). Data presented in the *y*-axis indicate genes expression as determined by qRT-PCR. * *p*-value < 0.05.

DE miRNA and DEGs were chosen in natal vs. E65 and adult vs. E65 to validate the sequencing data. In total, 11 DE miRNA and 14 DEGs were used to study with qRT-PCR. The results agreed with the sequencing data, showing that these two results authenticated each other (Figure 6 and Figure 7). Compared with E65, the upregulated genes were the *SLC2A4*, *ESR1* and *FBXO40,* while the downregulated genes were the *ASPN*, *WNT5A*, *KAZALD1* and *MASTL* in the natal, where the *SLC2A4*, *FBXO40*, *ASPN*, *WNT5A*, *KAZALD1* and *MASTL* showed significant differential expression. Moreover, the *KLF9* and *SLAMF7* were upregulated, whereas *SMAD5*, *WNT2*, *IGF1R*, *IGF2BP2* and *MDFI* were downregulated in the adult compared with E65, where *KLF9*, *WNT2* and *MDFI* showed significant differential expression.

For miRNA, compared with E65, the upregulated genes were the miR-128, miR133a-3p, miR-26a and miR-378, however, only miR-217 was downregulated in the natal, where miR-133a-3p showed significant differential expression. In addition, the miR-23a and miR-206 were upregulated, while the downregulated genes were miR-20b, miR-181c, miR-362 and miR-127 in the adult compared with E65. The miRNA and genes expression levels as measured with qRT-PCR were agreed with the sequencing data, which indicates the authenticity of RNA sequencing results.

## 4. Discussion

Deep sequencing approaches are the preferred biotechnology for prediction and identification of the novel genes and sRNA of livestock species. They are very suitable for studies in microbial genomics, oncology and other research including genomic analysis of rare cell populations. In this study, nine cDNA libraries and three sRNA libraries of LDM tissues were constructed and the expression patterns of mRNA and miRNA were identified in skeletal muscle of three developmental stages of Bamei pigs. By introducing the genome sequences of *Sus scrofa* as a reference, 24,045 genes containing 20,864 known and 3181 novel genes, and 329 miRNAs containing 301 known and 28 novel miRNAs were obtained.

In the current study, a total of 5388 DEGs were negatively regulated by 134 DE miRNA in three groups of pairwise comparisons. Most of these DEGs and miRNA were involved in the development and growth of muscles.

In the comparative analysis of natal and E65, genes MyoD family inhibitor (*MDFI*), asporin (*ASPN*) could be negatively regulated by miR-128. *MDFI* was enriched in “negative regulation of WNT signaling pathway”. High expression of *MDFI* inhibits porcine muscle satellite cell proliferation and promotes their differentiation [39]. *ASPN* was enriched in “anatomic structure development”. The rs41278695 SNP of *ASPN* has an effect on the cartilage extracellular matrix composition [40]. Wang et al. reported that miR-128 was DE in muscle tissues during four developmental stages (1, 30, 90 and 240 days of age) and it was validated by real-time quantitative PCR [41]. Our sequencing results are consistent with these studies and *MDFI*, *ASPN*, and miR-128 were also validated by qRT-PCR. All these results show that *ASPN* and *MDFI* could be regulated by miR-128 to participate in skeletal muscle growth. In addition, WNT family member 5A (*WNT5A*) which enriched in the “developmental process” was negatively regulated by miR-378 and miR-26a, *WNT5A*, miR-26a, and miR-378 were validated by qRT-PCR. Overexpression of WNT5A protein causes a sharp increase in satellite-cell proliferation [42]. The miR-26a was highly expressed during skeletal muscle developmental stages from 35 days post-coitus to postnatal 180 days and in LDM at postnatal 240 days of age [41,43,44]. The miR-378 participated in skeletal muscle development by regulating some genes in pigs [45].

The WNT family member 2 (*WNT2*) which enriched in the “developmental process” and SMAD family member 5 (*SMAD5*) could be negatively regulated by miR-23a in the comparative analysis of adult vs. E65. The qRT-PCR experiments of *WNT2*, *SMAD5*, and miR-23a were consistent with RNA-Seq results. The miR-23a was identified as an important regulator of skeletal muscle differentiation [46]. Hidestrand et al. reported that *WNT2* may be an important regulator of fibrosis in aged muscle [47]. In addition, has been also reported that *WNT2* was one of the top ten DEGs in skeletal muscle between healthy and splay leg piglets [48]. *SMAD5* participates in the BMP signals which enhance osteoblast differentiation and inhibit myogenic differentiation [49,50]. Hence, the miR-23a could be the major miRNA to indirectly regulate muscle differentiation through *WNT2* and *SMAD5* in pigs. Moreover, myogenic factor 6 (*MYF6*) was negatively regulated by miR-181c and miR-362 in the adult vs. E65. *MYF6* gene plays a key role during myoblast differentiation, and it transcriptionally and epigenetically determines the myogenic capacity of muscle progenitors [51]. The miR-181c was DE between the pigs at 63, 98 and 161 d of age [52] and it was one of the five miRNAs that showed diverse levels of methylation in their promoter regions between the *psoas major* and LDM muscle of Landrace pigs (the other four: miR-139 miR-378, miR-216 and miR-181d) [53]. The miR-362 was DE between E33, E65 and adult stages of porcine skeletal muscle tissues [54]. Combining the current results, *MYF6* gene, miR-181c and miR-362 play significant roles in muscle growth.

In the comparative analysis of adult vs. natal, insulin-like growth factor 1 receptor (*IGF1R*) could be negatively regulated by let-7a, miR-133a-3p and miR-7. Skeletal myogenic cells respond to the *IGF1* by differentiating or proliferating [55]. *IGF1R* gene as a receptor of *IGF1* is much important for skeletal myogenic cells and is essential during prenatal [56]. The let-7a is the most abundant miRNA in porcine muscle development, which has been widely reported [43,44,57,58]. Many previous studies found that miR-133a-3p is specifically expressed in skeletal muscle of pigs and it could promote skeletal muscle differentiation [41,59,60,61]. The miR-7 was DE amid different pigs breeds [62]. Hamrick et al. reported that the miR-7 expression levels were substantially enhanced with age in mouse muscle tissue [63]. Meanwhile, the let-7a negatively regulates kazal type serine peptidase inhibitor domain 1 (*KAZALD1*), insulin-like growth factor 2 mRNA binding protein 2 (*IGF2BP2*) and *IGF1R*. The *IGF1R*, *IGF2BP2* and *KAZALD1* were validated by qRT-PCR. The *IGF2BP2* (*IGF2* mRNA binding protein) always plays a vital role in the regulation of skeletal muscle growth and satellite cell activation [64]. *KAZALD1*, which is a DNA hyper-methylated gene, was particularly methylated in pleural mesothelioma and may act as prospective diagnostic markers [65]. The let-7a may also influence developmental processes by regulating *IGF1R*, *IGF2BP2* and *KAZALD1*.

To look for more reliable inverse miRNA–mRNA pairs, we searched miRNA target interaction data of pigs in mirTarBase [66]. Compared to the database, our results show that three pairs of mRNA- miRNA interactions (*DCX*-miR-204, *GNMT*-miR-296-3p and *CNN3*-miR-1) were concordant with the previous studies about miRNA and their experimentally validated targeted genes. The results suggest that these mRNA–miRNA interactions may have vital roles in the development and growth of muscles.

In addition to the genes listed above, we also observed many DEGs related to the development of muscles reported earlier. Gene activating transcription factor 3 (*ATF3*) and nitric oxide synthase 1 (*NOS1*) were DE between Iberian and Iberian and Duroc crossbred pigs [10]. Piórkowska et al. reported that Parvalbumin (*PVALB*), calcium and integrin-binding family member 2 (*CIB2*), choline phosphotransferase 1 (*CHPT1*), transcription factor Dp-2 (*TFDP2*) and breast cancer anti-estrogen resistance protein 3 (*BCAR3*) were DE amid pigs of Polish Landrace and Pulawska breeds [12]. These genes were also DEGs in our study. Meanwhile, many miRNA, such as miR-1, 127, 133b, 181a, 206, 214, 486, and 489 were found to be DE in other swine breeds [41,45,52,61].

The adipocytes are also related to the development and growth of muscle tissues. The skeletal muscle and brown fat are more related in lineage than white and brown fat [67]. Takada et al. reported that WNT signaling can inhibit adipogenesis [68]. There are some DEGs such as *WNT2*, *WNT2B*, *WNT3*, *WNT5A* and *WNT16* belonging to WNT pathway genes in our research and *WNT2* and *WNT5A* have been validated by qRT-PCR. These results demonstrate that WNT signaling genes are related to muscle growth in swine.

Our study expanded the recognition of miRNA in pig, especially in muscle tissue. Results from the current research indicated the advantage of next-generation sequencing and provided more details of miRNA expression in porcine muscle. Some miRNAs were validated in the present study. There were more miRNAs in the adult vs. E65 comparison than the other two groups, suggesting that adult vs. E65 may display greater alterations in physiological function. The present study has determined 134 DE miRNAs in the three developmental stages of the Bamei pig. The prediction of target genes of these DE miRNA with RNA-Seq data helped to identify the most important miRNA related to muscle development. Furthermore, the prospective target genes could be actively involved in the regulation of porcine muscle growth.

## 5. Conclusions

In conclusion, we obtained RNA-Seq and miRNA-Seq profiles of skeletal muscle from the different developmental stages of the Bamei pig, which are representative of major developmental stages of a pig. From these data, mRNA and miRNA that are DE amid the different developmental stages were identified by RNA and miRNA sequencing and qRT-PCR. Our outcomes provide significant information about miRNA and candidate genes associated with the development of porcine muscle and their possible role between different developmental stages. These findings will be useful and convenient for further studies on the development of muscles and molecular breeding.

## Supplementary Materials

The following are available online at https://www.mdpi.com/2076-2615/10/7/1198/s1, Table S1: The reports of RNA quality control, Table S2: Primers of DEGs used for the validation in analysis of qRT-PCR, Table S3: Primers of DE miRNA utilized for the validation in analysis of qRT-PCR, Table S4: The mRNA-Seq data filter and mapping summary, Table S5: Distribution of FPKM values of genes in nine samples, Table S6: The list of all DEGs with their corrected p-value and fold change. Table S7: The miRNA-Seq data filter and mapping summary, Table S8: The ratio of the mapped reads in sRNA libraries, Figure S1: The sequence length distribution of the miRNA in three samples (A: E65, B: natal and C: adult). Figure S2: Top 60 of GO enrichment for DE target genes from three groups of pairwise comparisons, Figure S3: Top 20 of KEGG pathways enrichment for DE target genes from three groups of pairwise comparisons.

## Abbreviations

The following abbreviations are used in this manuscript:

adult	10-month postnatal pigs
ASPN	Asporin
DEGs	DE genes
E65	65-day embryonic pigs
FDR	False discovery rate
FPKM	Fragments per kilobase of the exon model per million mapped reads
GO	Gene ontology
IGF1R	Insulin-like growth factor 1 receptor
IGF2BP2	Insulin-like growth factor 2 mRNA binding protein 2
KAZALD1	Kazal-type serine peptidase inhibitor domain 1
KEGG	Kyoto encyclopedia of genes and genomes
LDM	Longissimus dorsi muscle
MDFI	MyoD family inhibitor
MYF6	Myogenic factor 6
natal	0-day postnatal pigs
nt	Nucleotides
padj	Adjusted *p*-value
RIN	RNA integrity numbers
RNA-Seq	RNA sequencing
rRNA	Ribosomal RNA
SMAD5	SMAD family member 5
snRNA	Small nuclear RNA
snoRNA	Small nucleolar RNA
sRNA	Small RNA
TPM	Transcript per million
tRNA	Transfer RNA
vs.	Versus
WNT2	Wnt family member 2
WNT5A	Wnt family member 5A
