# Supplementary material for: Analysis of Transcriptome and miRNAome in the Muscle of Bamei Pigs at Different Developmental Stages
Source: Animals (Basel). 2020 Jul 15;10(7):1198. doi: 10.3390/ani10071198 (PMC7401622; doi:10.3390/ani10071198)
Supplement: Supplementary file 1 [file animals-10-01198-s001.zip › animals-667177-supplementary/Supplementary Figure S2.pdf]

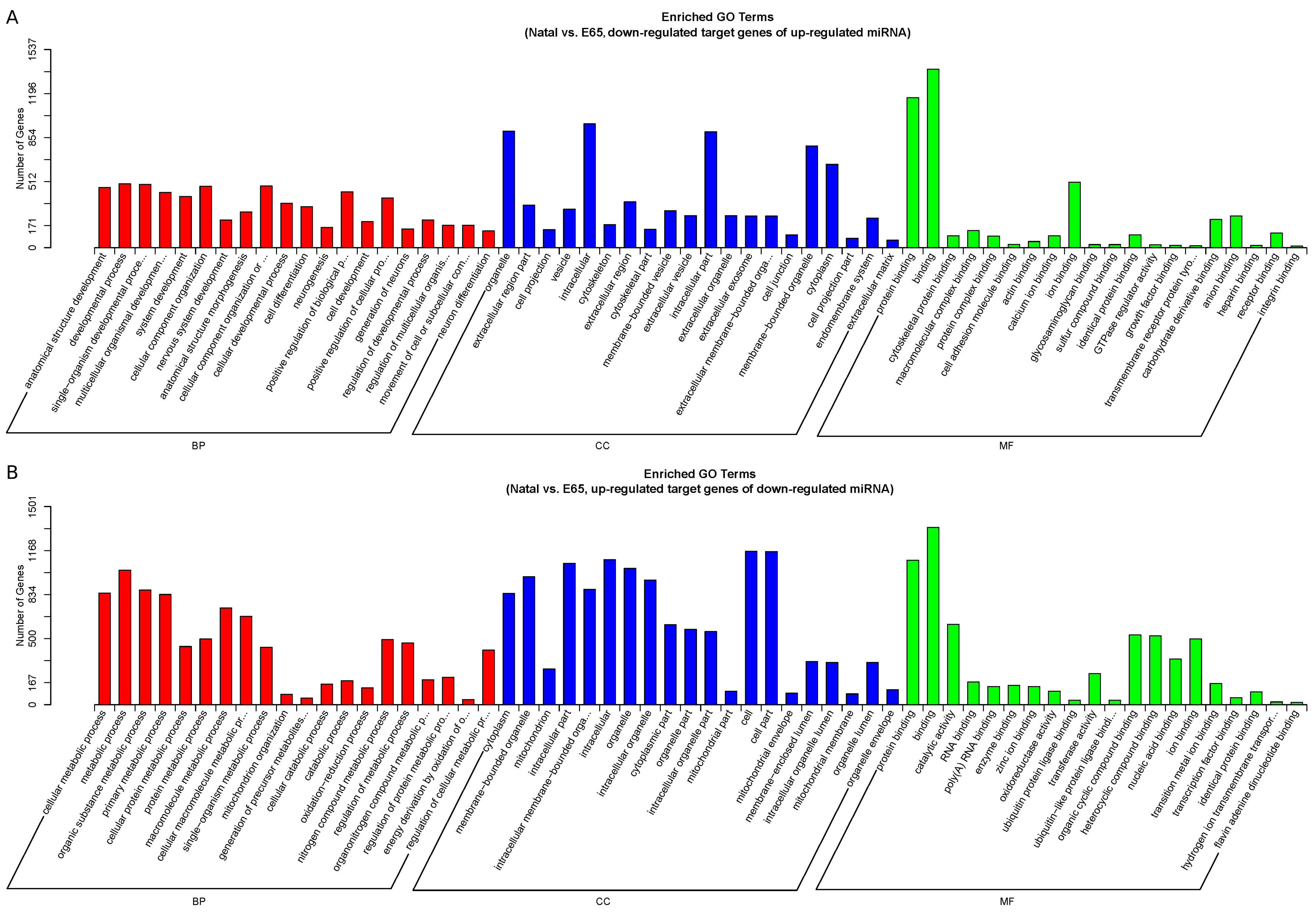
A
Enriched GO Terms
(Natal vs. E65, down-regulated target genes of up-regulated miRNA)
Number of Genes
0
171
512
854
1196
1537
anatomical structure development
developmental process
single−organism developmental proce...
multicellular organismal developmen...
system development
cellular component organization
nervous system development
anatomical structure morphogenesis
cellular component organization or ...
cellular developmental process
cell differentiation
neurogenesis
positive regulation of biological p...
cell development
positive regulation of cellular pro...
generation of neurons
regulation of developmental process
regulation of multicellular organis...
movement of cell or subcellular com...
neuron differentiation
organelle
extracellular region part
cell projection
vesicle
intracellular
cytoskeleton
extracellular region
cytoskeletal part
membrane−bounded vesicle
extracellular vesicle
intracellular part
extracellular organelle
extracellular exosome
extracellular membrane−bounded orga...
cell junction
membrane−bounded organelle
cytoplasm
cell projection part
endomembrane system
extracellular matrix
protein binding
binding
cytoskeletal protein binding
macromolecular complex binding
protein complex binding
cell adhesion molecule binding
actin binding
calcium ion binding
ion binding
glycosaminoglycan binding
sulfur compound binding
identical protein binding
GTPase regulator activity
growth factor binding
transmembrane receptor protein tyro...
carbohydrate derivative binding
anion binding
heparin binding
receptor binding
integrin binding
BP
CC
MF
B
Enriched GO Terms
(Natal vs. E65, up-regulated target genes of down-regulated miRNA)
Number of Genes
0
167
500
834
1168
1501
cellular metabolic process
metabolic process
organic substance metabolic process
primary metabolic process
cellular protein metabolic process
protein metabolic process
macromolecule metabolic process
cellular macromolecule metabolic pr...
single−organism metabolic process
mitochondrion organization
generation of precursor metabolites...
cellular catabolic process
catabolic process
oxidation−reduction process
nitrogen compound metabolic process
regulation of metabolic process
organonitrogen compound metabolic p...
regulation of protein metabolic pro...
energy derivation by oxidation of o...
regulation of cellular metabolic pr...
cytoplasm
membrane−bounded organelle
mitochondrion
intracellular part
intracellular membrane−bounded orga...
intracellular
organelle
intracellular organelle
cytoplasmic part
organelle part
intracellular organelle part
mitochondrial part
cell
cell part
mitochondrial envelope
membrane−enclosed lumen
intracellular organelle lumen
mitochondrial membrane
organelle lumen
organelle envelope
protein binding
binding
catalytic activity
RNA binding
poly(A) RNA binding
enzyme binding
zinc ion binding
oxidoreductase activity
ubiquitin protein ligase binding
transferase activity
ubiquitin−like protein ligase bindi...
organic cyclic compound binding
heterocyclic compound binding
nucleic acid binding
ion binding
transition metal ion binding
transcription factor binding
identical protein binding
hydrogen ion transmembrane transpor...
flavin adenine dinucleotide binding
BP
CC
MF

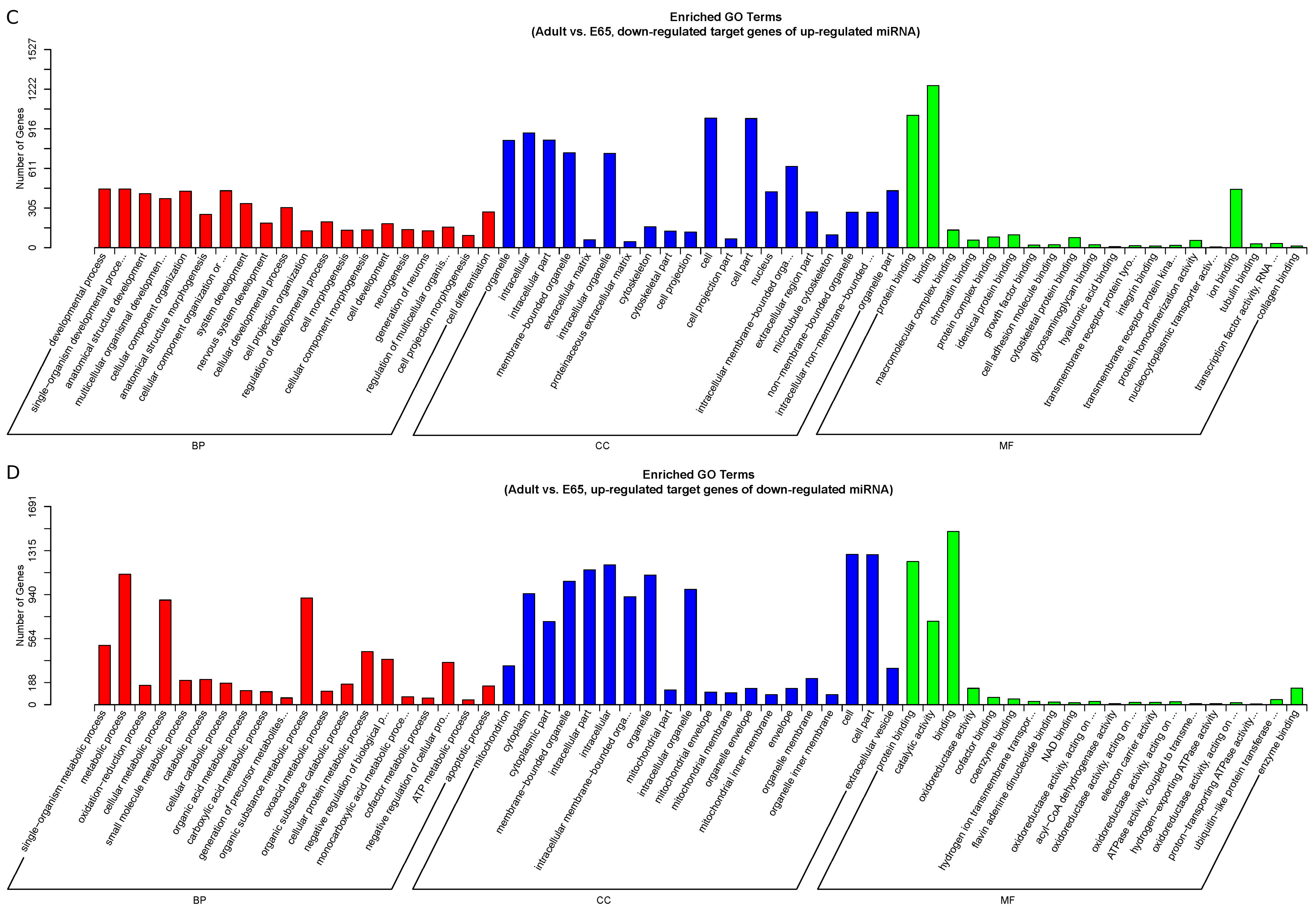
C
Enriched GO Terms
(Adult vs. E65, down-regulated target genes of up-regulated miRNA)
Number of Genes
0
305
611
916
1222
1527
developmental process
single−organism developmental proce…
anatomical structure development
multicellular organismal developmen…
cellular component organization
anatomical structure morphogenesis
cellular component organization or …
system development
nervous system development
cellular developmental process
cell projection organization
regulation of developmental process
cell morphogenesis
cellular component morphogenesis
cell development
neurogenesis
generation of neurons
regulation of multicellular organis…
cell projection morphogenesis
cell differentiation
organelle
intracellular
intracellular part
membrane−bounded organelle
extracellular matrix
intracellular organelle
proteinaceous extracellular matrix
cytoskeleton
cytoskeletal part
cell projection
cell
cell projection part
cell part
nucleus
intracellular membrane−bounded orga…
extracellular region part
microtubule cytoskeleton
non−membrane−bounded organelle
intracellular non−membrane−bounded …
organelle part
protein binding
binding
macromolecular complex binding
chromatin binding
protein complex binding
identical protein binding
growth factor binding
cell adhesion molecule binding
cytoskeletal protein binding
glycosaminoglycan binding
hyaluronic acid binding
transmembrane receptor protein tyro…
integrin binding
transmembrane receptor protein kina…
protein homodimerization activity
nucleocytoplasmic transporter activ…
ion binding
tubulin binding
transcription factor activity, RNA …
collagen binding
BP
CC
MF
D
Enriched GO Terms
(Adult vs. E65, up-regulated target genes of down-regulated miRNA)
Number of Genes
0
188
564
940
1315
1691
single−organism metabolic process
metabolic process
oxidation−reduction process
cellular metabolic process
small molecule metabolic process
catabolic process
cellular catabolic process
organic acid metabolic process
carboxylic acid metabolic process
generation of precursor metabolites…
organic substance metabolic process
oxoacid metabolic process
organic substance catabolic process
cellular protein metabolic process
negative regulation of biological p…
monocarboxylic acid metabolic proce…
cofactor metabolic process
negative regulation of cellular pro…
ATP metabolic process
apoptotic process
mitochondrion
cytoplasm
cytoplasmic part
membrane−bounded organelle
intracellular part
intracellular
intracellular membrane−bounded orga…
organelle
mitochondrial part
intracellular organelle
mitochondrial envelope
mitochondrial membrane
organelle envelope
mitochondrial inner membrane
envelope
organelle membrane
organelle inner membrane
cell
cell part
extracellular vesicle
protein binding
catalytic activity
binding
oxidoreductase activity
cofactor binding
coenzyme binding
hydrogen ion transmembrane transpor…
flavin adenine dinucleotide binding
NAD binding
oxidoreductase activity, acting on …
acyl−CoA dehydrogenase activity
oxidoreductase activity, acting on …
electron carrier activity
oxidoreductase activity, acting on …
ATPase activity, coupled to transme…
hydrogen−exporting ATPase activity
oxidoreductase activity, acting on …
proton−transporting ATPase activity…
ubiquitin−like protein transferase …
enzyme binding
BP
CC
MF

E

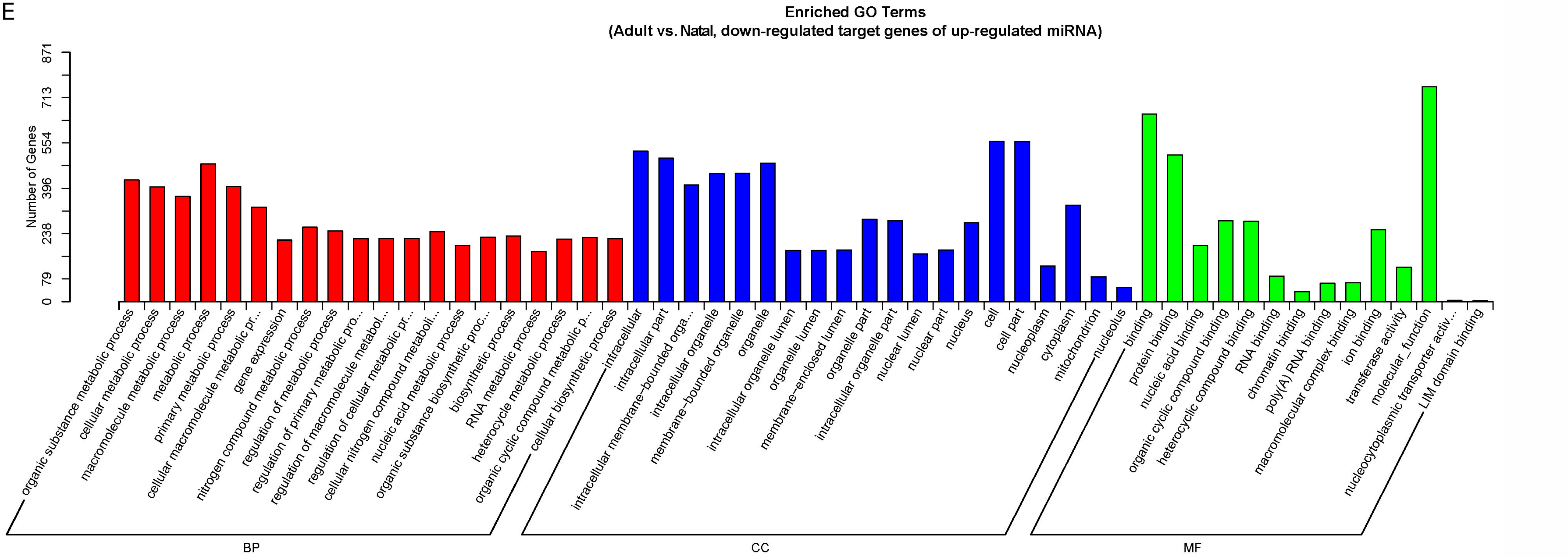
Enriched GO Terms
(Adult vs. Natal, down-regulated target genes of up-regulated miRNA)
Number of Genes
0
79
238
396
554
713
871
organic substance metabolic process
cellular metabolic process
macromolecule metabolic process
metabolic process
primary metabolic process
cellular macromolecule metabolic pr…
gene expression
nitrogen compound metabolic process
regulation of metabolic process
regulation of primary metabolic pro…
regulation of macromolecule metabol…
regulation of cellular metabolic pr…
cellular nitrogen compound metaboli…
nucleic acid metabolic process
organic substance biosynthetic proc…
biosynthetic process
RNA metabolic process
heterocycle metabolic process
organic cyclic compound metabolic p…
cellular biosynthetic process
intracellular
intracellular part
intracellular membrane−bounded orga…
intracellular organelle
membrane−bounded organelle
organelle
intracellular organelle lumen
organelle lumen
membrane−enclosed lumen
organelle part
intracellular organelle part
nuclear lumen
nuclear part
nucleus
cell
cell part
nucleoplasm
cytoplasm
mitochondrion
nucleolus
binding
protein binding
nucleic acid binding
organic cyclic compound binding
heterocyclic compound binding
RNA binding
chromatin binding
poly(A) RNA binding
macromolecular complex binding
ion binding
transferase activity
molecular_function
nucleocytoplasmic transporter activ…
LIM domain binding
BP
CC
MF

F

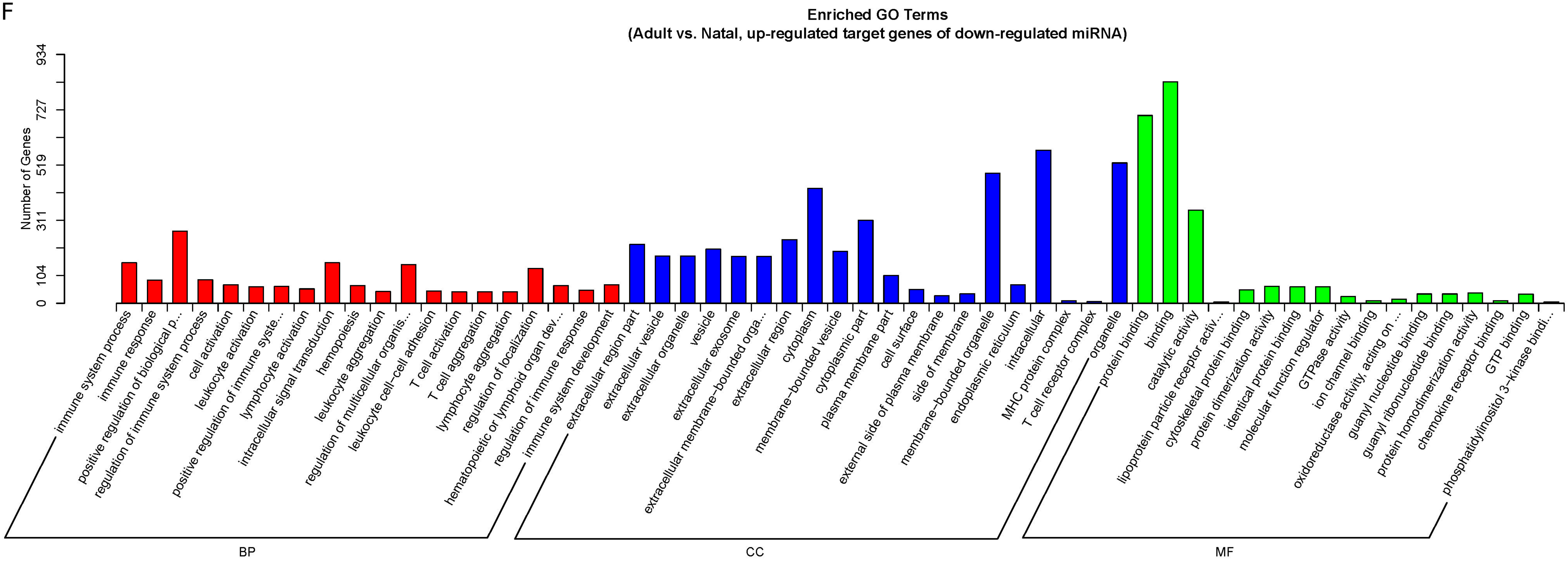
Enriched GO Terms
(Adult vs. Natal, up-regulated target genes of down-regulated miRNA)
Number of Genes
0
104
311
519
727
934
immune system process
immune response
positive regulation of biological p…
regulation of immune system process
cell activation
leukocyte activation
positive regulation of immune syste…
lymphocyte activation
intracellular signal transduction
hemopoiesis
leukocyte aggregation
regulation of multicellular organis…
leukocyte cell−cell adhesion
T cell activation
T cell aggregation
lymphocyte aggregation
regulation of localization
hematopoietic or lymphoid organ dev…
regulation of immune response
immune system development
extracellular region part
extracellular vesicle
extracellular organelle
vesicle
extracellular exosome
extracellular membrane−bounded orga…
extracellular region
cytoplasm
membrane−bounded vesicle
cytoplasmic part
plasma membrane part
cell surface
external side of plasma membrane
side of membrane
membrane−bounded organelle
endoplasmic reticulum
intracellular
MHC protein complex
T cell receptor complex
organelle
protein binding
binding
catalytic activity
lipoprotein particle receptor activ…
cytoskeletal protein binding
protein dimerization activity
identical protein binding
molecular function regulator
GTPase activity
ion channel binding
oxidoreductase activity, acting on …
guanyl nucleotide binding
guanyl ribonucleotide binding
protein homodimerization activity
chemokine receptor binding
GTP binding
phosphatidylinositol 3−kinase bindi…
BP
CC
MF
