# Supplementary figures and images for: Analysis of Transcriptome and miRNAome in the Muscle of Bamei Pigs at Different Developmental Stages
Source: Animals (Basel). 2020 Jul 15;10(7):1198. doi: 10.3390/ani10071198 (PMC7401622; doi:10.3390/ani10071198)

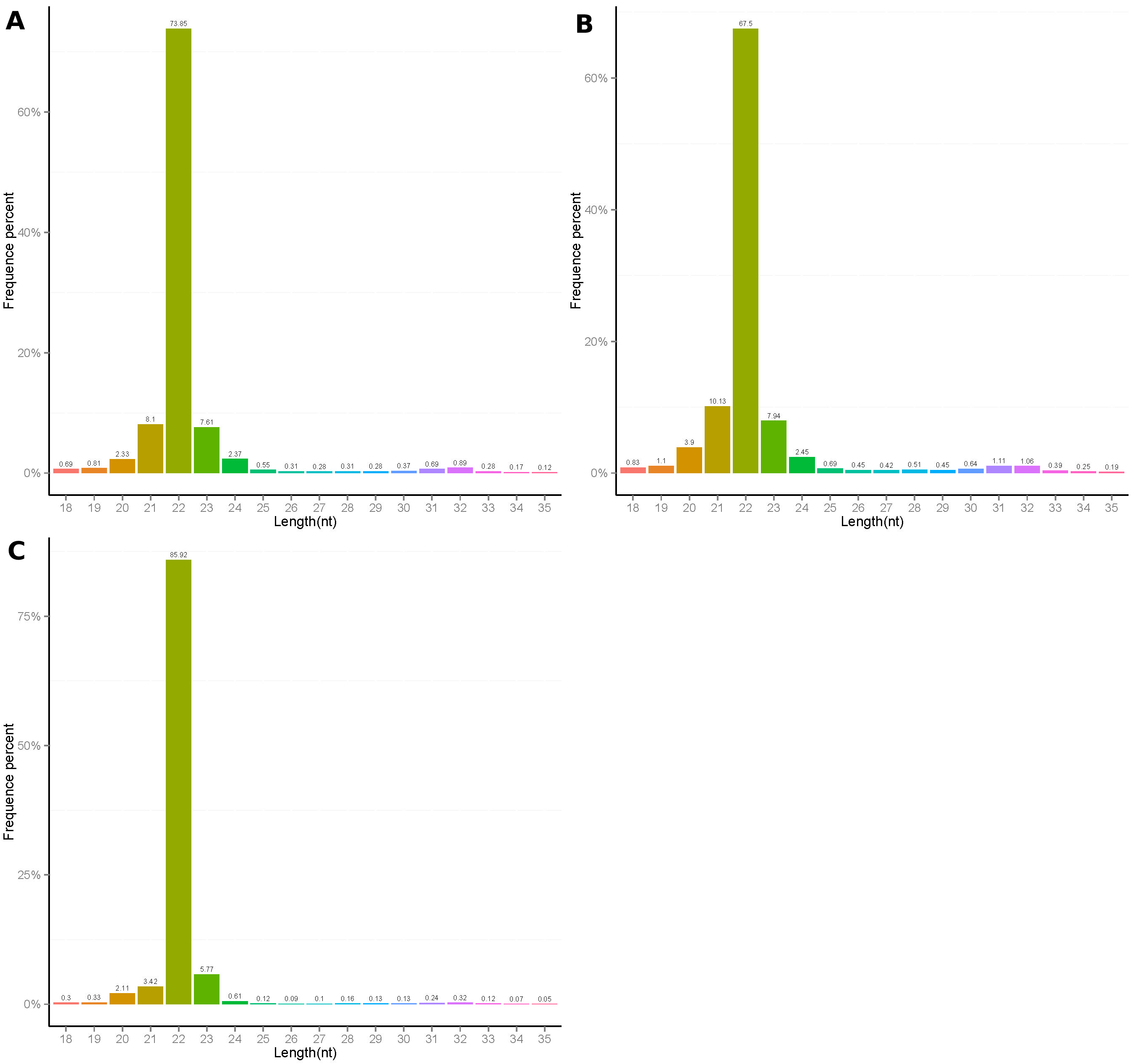

Supplement: Supplementary file 1 [file animals-10-01198-s001.zip › animals-667177-supplementary/Supplementary Figure S1.tif]

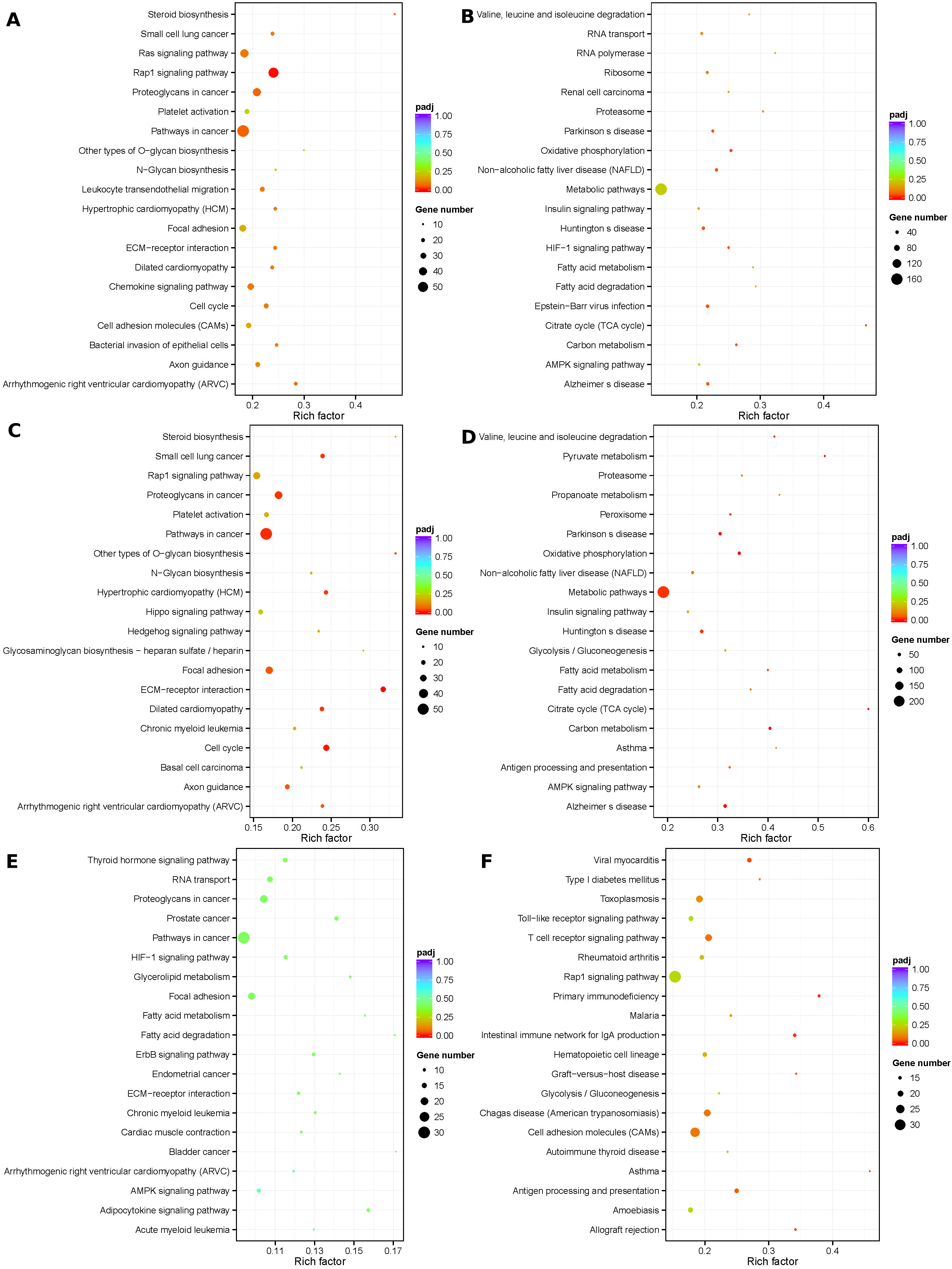

Supplement: Supplementary file 1 [file animals-10-01198-s001.zip › animals-667177-supplementary/Supplementary Figure S3.tif]
